# Folate deficiency in an unselected population in Calgary, Alberta and its relationship with red blood cell macrocytosis

**DOI:** 10.1186/s13104-015-1273-y

**Published:** 2015-07-25

**Authors:** Matthew Budd, Christopher Naugler

**Affiliations:** Calgary Laboratory Services, Calgary, Canada; Department of Pathology and Laboratory Medicine, University of Calgary, C410, Diagnostic and Scientific Centre, 3535 Research Road NW, Calgary, AB T2L 2K8 Canada; Department of Family Medicine, University of Calgary, C410, Diagnostic and Scientific Centre, 3535 Research Road NW, Calgary, AB T2L 2K8 Canada

**Keywords:** Folate, Macrocytosis, Receiver operator characteristic curve, Demographics, Canada

## Abstract

**Background:**

Folate deficiency is rare in western countries and therefore blood tests for folate level have limited indications. One such indication is red cell macrocytosis, however it is unclear if the association of macrocytosis with folate deficiency is robust enough to serve as a risk marker. Our objective is to determine whether macrocytosis is a useful marker for folate deficiency.

**Findings:**

Paired data from the Calgary Laboratory Services Information System was analyzed using receiver operating characteristic (ROC) curves to determine strength of association between mean corpuscular volume and serum folate. Strength of association was analyzed for serum folate cut-off values of 12, 10, 8, and 6 nmol/L. Overall, 0.2% of individuals were folate deficient (<6 nmol/L serum folate). Based on ROC curves, at each cut-off level, mean corpuscular volume was a poor predictive marker for serum folate level.

**Conclusions:**

Folate deficiency is rare in Calgary, Alberta. Macrocytosis is not a strong predictor for folate deficiency.

## Findings

In the late 1990s, Canadian and US federal governments mandated folic acid fortification of all commercial wheat and cereal-grain products, in the amount of 140 μg folate/g [1]. Adherence to this mandate was nearly universal by January of 1998 [2], resulting in an estimated 44–64% increase in mean population red blood cell (RBC) folate concentration and a 119–161% increase in serum folate concentration [3].

Prior to fortification, population estimates for RBC folate deficiency were as high as 38%, and 16% for serum folate deficiency [4]. Increased risk of folate deficiency has been observed in elderly populations, low-income and non-white populations [5], and Aboriginal cultures [6]. However, recent studies have concluded that fortification has resulted in the near-elimination of folate deficiency-related anemia in Canada [7, 8]. Despite this, tests for serum folate levels are still commonly ordered by Canadian physicians.

The purpose of this study is twofold: firstly, to document the rate of folate deficiency among serum folate tests ordered in Calgary, Alberta; and secondly, to determine whether red cell mean corpuscular volume (MCV) can reliably be used as a marker for folate deficiency. Macrocytosis associated with megaloblastic anemia has been tied to both B12 and folate deficiency [9], which has prompted questions regarding the necessity of folate testing for patients that do not exhibit anemia-related macrocytosis.

This research was approved by the University of Calgary Conjoint Research Ethics Board (ID: REB 13-0376). We examined data on paired folate levels and MCV in the Calgary Laboratory Services Laboratory (CLS) Information System database for the period of 01 January 2010 to 30 September 2013. CLS is the sole provider of laboratory testing for the Calgary Health Region in Southern Alberta (catchment population of 1.4 million people). The extracted data included values for vitamin B12, MCV, and serum folate level. RBC folate was rarely measured and was not considered in this study. As we were interested only in the association of MCV and folate level, we included only patients with a B12 level in the normal range for our laboratory (greater than 155 pmol/L).

Receiver operating characteristic (ROC) curves were used to analyze the association between MCV and serum folate values, with the area under the curve (AUC) representing the strength of this correlation. An AUC of 0.8 is considered a strong correlation (the theoretical maximum value being 1), while an AUC in the 0.5–0.6 range is typically indicative of a non-predictive or random distribution [10]. We performed the AUC analysis for four threshold values for serum folate: <12 nmol/L (the lower limit of normal for Alberta Health Services), <6 nmol/L (the level considered deficient by Colaptino et al. [7]), and two intermediate values of <10 and <8 nmol/L. For every serum folate level we looked for an MCV value tested within ±7 days. To avoid pseudoreplication, only one (the first in this date range) paired folate-MCV test was included for each individual. To avoid a possible bias of macrocytosis due to vitamin B12 deficiency, all individuals with a vitamin B12 level <155 pmol/L were excluded from the analysis.

**Fig. 1:**
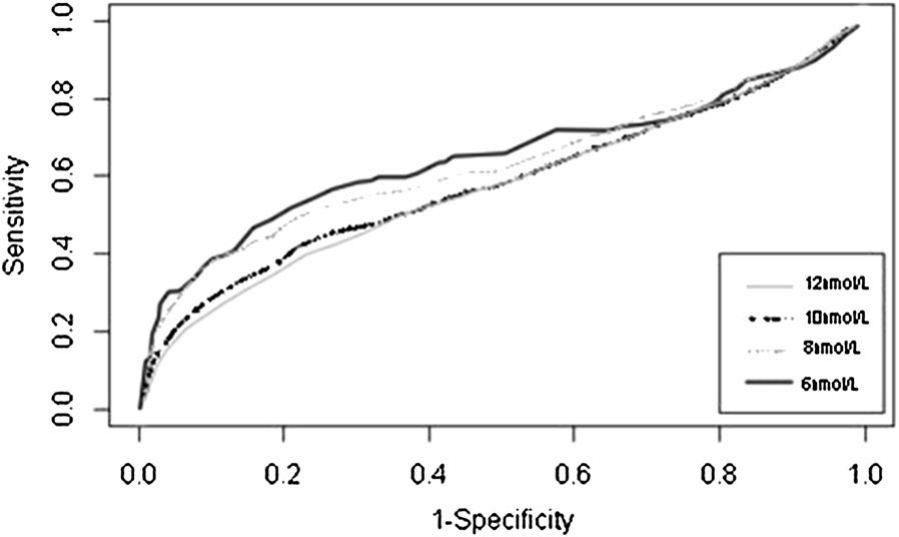
Receiver operator characteristic curves for red blood cell mean corpuscular volume (MCV) and serum folate deficiency using the cut-off values of <12, <10, <8, and <6 nmol/L for serum folate. All cut-off values show a poor relationship between MCV and folate deficiency (areas under the curve are given in the *text*).

Paired folate and MCV vales were available for a total of 46,827 individuals (27,021 women and 19,805 men). For the purposes of analysis folate values reported as >45.4 nmol/L (the upper limit of our reporting range) were converted to 45.4 nmol/L. The mean age was 56.9 and the mean serum folate level was 33.2 nmol/L. 3.6% of individuals had a folate value of <12 nmol/L (the lower normal range in Alberta laboratories). However, for a folate cutoff value of <6 nmol/L—the cutoff for deficiency used by Colapinto et al. [7]—only 0.2% of individuals were considered deficient. The ROC analysis showed that for a threshold value of <12 nmol/L serum folate compared against MCV, the AUC was 0.575; for a threshold value of 10 nmol/L serum folate, the AUC was 0.582; for a threshold value of 8 nmol/L serum folate, the AUC was 0.626; finally, for a threshold value of 6 nmol/L serum folate, the AUC was 0.613 (Fig. 1). Finally, we examined the potential contributions of age, sex, hemoglobin level and ferritin level (as a measure of co-existing iron deficiency) using a binary logistic regression. We conducted this analysis on a subset of 22,347 individuals for which full data was available. The results are shown in Table 1. This analysis showed that men were more likely to be macrocytic than women (OR 0.788) and lower folate levels were modestly associated with macrocytosis (OR 0.99). Hemoglobin was not associated with macrocytosis in this model and Vitamin B12 level, age and ferritin, although statistically significant were associated with odds ratios close to 1. The lack of association with vitamin B12 level was not surprising as B12 deficiency was an exclusion criteria for the study.

**Table 1:** Binary logistic regression model showing the association of independent associations of serum folate level, hemoglobin level, vitamin B12 level, age, ferritin level, and sex with the presence of RBC macrocytosis (MCV >100)

Variable	P value	OR (95% CI)
Serum folate level	<0.001	0.990 (0.986–0.984)
Hemoglobin level	0.141	0.999 (0.999–1.004)
Vitamin B12 level	<0.001	1.000 (1.000–1.001)
Age	<0.001	1.023 (1.021–1.026)
Serum ferritin level	<0.001	1.001 (1.001–1.001)
Sex (female)	0.004	0.788 (0.788–0.957)

Using a cutoff value of <6 nmol/L we observed a deficiency rate of 0.2%. This is 20% of the deficiency rate reported in a previous Canadian study [7]. The reason for this is unclear. It may be due to either a lower deficiency rate within Calgary as compared to the Canadian population at large, or it may be due to a mismatch between testing effort and at risk individuals.

None of the AUC values we obtained for the four comparisons of MCV and serum folate suggested a strong level of association. Therefore, despite the well-known association between folate deficiency and macrocytosis, our results indicate that macrocytosis is at best a weak marker for folate deficiency and cannot be used to screen for folate deficiency.

There are several limitations to this study. First of all, as we did not use a random sample of individuals but rather considered all tests done at our lab, the prevalence we report cannot be directly compared with previous work. Further work is needed to distinguish between these possibilities. A second limitation is that other than vitamin B12 deficiency, we did not have data on other possible causes of macrocytosis such as medications and myeloproliferative disorders. We did not have data on levels of methylmalonic acid or homocysteine which may have indicated occult vitamin B12 or folate deficiency. It is possible that other causes of macrocytosis could have weakened any relationship in our data.

Taken in context with other recent work on folate deficiency in Canada, our results indicate that while folate deficiency is rare in Canada, macrocytosis is not a useful marker for its presence.

## Abbreviations

RBC: red blood cell
MCV: mean corpuscular volume
ROC: receiver operator characteristic
AUC: area under the curve
